# Absence of Multiple Sclerosis and Demyelinating Diseases among Lacandonians, a Pure Amerindian Ethnic Group in Mexico

**DOI:** 10.1155/2012/292631

**Published:** 2012-08-29

**Authors:** Jose Flores, Silvia González, Ximena Morales, Petra Yescas, Adriana Ochoa, Teresa Corona

**Affiliations:** ^1^Neurodegenerative Diseases Laboratory, The National Institute of Neurology and Neurosurgery, Insurgentes Sur 3877 Col. La Fama. Del. Tlalpan, CP 14269, Mexico City 14000, DF, Mexico; ^2^Genetics Laboratory, The National Institute of Neurology and Neurosurgery, Insurgentes Sur 3877 Col. La Fama. Del. Tlalpan, CP 14269, Mexico City, DF, Mexico

## Abstract

Multiple Sclerosis (MS) is a highly polymorphic disease characterized by different neurologic signs and symptoms. In MS, racial and genetic factors may play an important role in the geographic distribution of this disease. Studies have reported the presence of several protective alleles against the development of autoimmune disorders. In the case of MS, however, they help define MS as a complex disease, and confirm the importance of environmental agents as an independent variable not associated with ethnicity. We carried out an on-site epidemiological study to confirm the absence of MS or NMO among Lacandonians, a pure Amerindian ethnic group in Mexico. We administered a structured interview to 5,372 Lacandonians to assess by family background any clinical data consistent with the presence of a prior demyelinating event. Every participating subject underwent a comprehensive neurological examination by a group of three members of the research team with experience in the diagnosis and treatment of demyelinating disorders to detect clinical signs compatible with a demyelinating disease. We did not find any clinical signs compatible with multiple sclerosis among study participants.

## 1. Introduction

MS is a highly polymorphic disease characterized by diverse neurological signs and symptoms (e.g., optical neuritis, dizziness, disturbances in bladder control, peripheral sensory neuropathies, and/or limb weakness). In most patients (80%) MS has a relapse-remitting pattern, whereas in a minority it can be primary progressive (6%), secondary progressive (10%), or progressive relapsing (4%).

MS incidence rates vary significantly depending on geographic location and ethnic origin, ranging from 1 : 500 in Northern Europe to 1 : 100,000 in tropical countries [1]. Individuals migrating from a tropical region to Northern Europe seem to maintain a low risk. Although a predisposition to develop MS may be hereditary, environmental factors display a significant interaction with genetic factors to help determine disease outcome. An effect of shared environment has not been yet proven, and little is known about potential environmental triggers (e.g., viral infections, including Epstein-Barr or Varicella Zoster virus) [2]. 

Genes associated with a predisposition to develop MS are being actively sought following two main approaches: broad genomic screenings and targeted gene searches. Over 15 research groups have reported linkages of chromosomal regions with MS: chromosomes 6p (on which HLA is located), 5p, 5q, 17q, and 19q [3–5]. However more than half of the human genome has been linked to MS to varying degrees. An association with the *HLA-DRB1∗1501-DQB1∗0602* haplotype has been repeatedly found in high-risk, Northern European populations. About 25% to 30% of monozygotic twins of an affected individual develop MS compared with only 3% to 5% of dizygotic twins and other siblings [4, 5]. 

The GAMES consortium recently examined 6,000 microsatellite markers among thousands of samples. Although regions in chromosome 1q43 could be consistently associated with MS, other findings were difficult to replicate. Independent genome-screening results also point to the 1q43 chromosomal region (LOD score >3 using nonparametric multipoint analysis) as a genetic risk factor for MS, as reported by the US and French MS Genetics Groups after analyzing data sets from 186 multiplex families [6].

Gene polymorphisms in the apolipoprotein E (*APOE*) gene of patients with MS have been reported by most as not associated with an increased risk of developing MS, but different groups found a discordant effect of this locus on the severity and/or disease progression in individuals already affected with MS [1, 4]. 

Reviewing epidemiological literature can help us identify the main demographic and sociocultural features of MS among ethnic groups. Ethnic groups differ depending on three variables: race, religion, and nationality. Although MS has been observed in the three main racial groups worldwide (white Caucasian, oriental, and black), the disease tends to be unequally distributed. Two hypotheses have been put forth to help explain the unequal susceptibility of MS among races. The first hypothesis states that whereas the highest MS rates are found in regions of the world inhabited largely by white populations, the lowest rates tend to be found in those areas where nonwhites live. The second hypothesis suggests that racially different groups living in the same geographical area tend to have different rates of MS, although there is a tendency for whites to display MS more often than nonwhites. Data associated with the first and second hypotheses support a uniformly higher and lower risk for MS among whites and nonwhites, respectively. If these findings are in fact valid, these studies indicate that racial (genetic) factors may play an important role in the distribution of MS. Another view on the geographic diversity of MS indicates the existence of protective alleles that help against to develop autoimmune disorders [7, 8]. The latter view seems to indicate MS is an acquired, exogenous (environmental) disease and confirms the importance of an environmental agent in disease causality that seems to be independent of ethnicity. 

We carried out an on-site epidemiological study among Lacandonians, a pure ethnic group in Mexico to describe the presence or absence of clinical data compatible with MS or NMO.

## 2. Materials and Methods

We carried out an on-site epidemiological study to confirm if Lacandonians, a pure Amerindian ethnic group in Mexico, had clinical signs and symptoms compatible with MS or with other demyelinating diseases. Our research team visited the Lacandonian forest located in the southern state of Chiapas, a south-west Mexican state with nearly 4 million of habitants; 52% lives in rural area. 12 of 62 indigenous populations in Mexico live there, Tseltal, Tsotsil, Ch′ol, Tojol-ab′al, Zoque, Chuj, Kanjobal, Mam, Jacalteco, Mochó, Cakchiquel y Lacandón o Maya Caribe; all these people conform near 1 million habitants; Lacandonians are nearly 50,000 people divided in different areas in Lacandonian forest; the main communities where Lacandonians live are Nahá and Metzaboc in the north and Lacan Há Chan Sayab, Bethel and El Tumbo in the south, a region throughout all year has 20–29°C, humid tropical weather. We interviewed the community leader and we explained the nature of the study and requested informed consent. We also sought help from local general practitioners working in different communities (Nahá and Metzaboc, two communities located in the northern part and Lacan Há Chan Sayab, Bethel and El Tumbo, in the south). Our study instrument included a survey with basic socio-demographic characteristics, a structured interview, and a comprehensive neurological examination. The research team concentrated on two strategic points in the north and south areas of the state where Lacandonians often gather (Nahá and Lacan Há Chan Sayab).

A group of local translators helped the research team to administer the study instrument, and during the face-to-face interview, we mention that near of 85% of all Lacandonians speak mother language and Spanish, so the translator was used only in few cases. The ad hoc clinical questionnaire was designed by senior members of our team adapted from the standardized instrument routinely used at the National Institute of Neurology and Neurosurgery, a tertiary care referral center in Mexico City. The study instrument was administered by a group of 50 general local practitioners previously trained in study protocol with the help of community translators. We included a sample of 5,372 Lacandonians in our study. Each participant gave oral or written informed consent with prior authorization from the community leader. We explained to all study participants we were interested in exploring the presence or absence of clinical data suggestive of a previous demyelinating event. All study participants were given a thorough neurological exam performed by senior neurologists specializing in demyelinating disorders. The exam emphasized the presence of any clinical sign suggestive of a demyelinating disease.

During the-face-to face interview, and after consent for biological samples from participants was obtained, we collected blood samples (30 mls by venopuncture) to perform further genetic analysis that would substantiate our epidemiological findings. By the nature of study we do not calculate a power sample size.

## 3. Results

We collected clinical and demographic data from 5,372 Lacandonians living in five communities in Chiapas, Mexico. We collected 350 DNA blood samples for future analysis and during our first screening identified that 99% of samples were O+ blood type.

The male/female ratio was 1.2 : 1. 1,771 (32%) were between 15 and 60 years. We were not able to identify by family background or clinical history the presence of any demyelinating disease in our sample. The main three causes of disease among our sample were acute respiratory disease, gastrointestinal disease, and conjunctivitis.  Table 1 describes the ten most common causes of attention during our visit. We did not identify patients with symptoms of MS or neuromyelitis optica (NMO).

## 4. Discussion

MS prevalence is higher among northern Europeans and Americans who report European ancestry or have a predominantly Caucasian extraction than among Native Americans living in the same latitudes. Consensus from the Latin American Committee for Treatment and Research in Multiple Sclerosis (LACTRIMS) [9, 10] suggests that nonmixed Native Americans or Amerindians are seldom affected by MS. The prevalence of MS continues to increase among Mestizos, who have a complex mixture of Caucasian and Mongoloid genes and constitute the core people living in Latin America. Studiesamong ethnic groups considered *pure* such as the Tarahumara, Pima, Mazahua, and Quarijo Indian groups [9–11] in Chihuahua, a northern state in Mexico, or among ethnic groups living in the central regions, of Mexico such as the Nahuatl, Mexica, Huastecos, or Otomies do not seem to report MS. The disease has not been reported among other American Indian groups such as the Aymars in Perú, the Xingu or among the Yanomamis from Brazil, or the Mapuche from Chile [9, 10].

It seems plausible that American Indians are protected against MS or other demyelinating disorders like Neuromelitis Optica (NMO) in part due to their ancestral genetic profile. In our study using a large sample of Lacandonian subjects who underwent comprehensive clinical and neurological testing, we did not find any clinical evidence compatible with any demyelinating disorder. Other studies done among mestizos in Mexico have shown that MS mestizo (mixture of indigenous and Spanish descent) patients share an HLA-DRB1 profile similar to the one found among European groups at high risk for MS. This finding was not corroborated in our study among ethnically pure groups such as the Lacandonians who display a low frequency of these haplotypes thereby pointing towards a genetic admixture between Caucasian and Amerindians, a mixture that took place over the last five centuries when Spain conquered most of the region [12]. Not only is the genetic background of Latin America singular, but also the exposure to certain infectious agents may be a critical factor that may help prevent or ameliorate some autoimmune diseases such as MS. A dichotomous association has been shown to exist between the global distribution of MS and the presence of parasitic infections [13, 14]. In the case of our study participants, Lacandonians displayed a high frequency of gastrointestinal disorders that may indicate that parasitic diseases could be an epidemiological finding that stands in contrast with developing any type of demyelinating disorders.

## 5. Conclusion

We did not find clinical evidence of MS or NMO among our sample of Lacandonians, a finding that may indicate a likely environmental and genetic background that influences the incidence of these types of autoimmune disorders.

## Figures and Tables

**Table 1:** Main ten causes of medical attention during the visit.

Number	Diagnosis	Nahá	El Tumbo	Lacanjá Chansayab	Total
1	Acute respiratory disease	89	83	27	199
2	Gastrointestinal disease	21	35	0	56
3	Conjunctivitis	10	5	0	15
4	Dermatosis	20	14	0	34
5	Parasitosis	75	73	9	157
6	Sexually transmitted infections	0	0	0	0
7	Cervical vaginitis	42	32	11	85
8	Fever unknown origin	0	6	0	6
9	Urinary tract infections	12	21	15	48
10	Other	336	198	160	694

	Total	605	467	222	1294
